# What Might Be Required for Inspections to Be Considered Fair? Comment on "What Lies Behind Successful Regulation? A Qualitative Evaluation of Pilot Implementation of Kenya’s Health Facility Inspection Reforms"

**DOI:** 10.34172/ijhpm.2022.7296

**Published:** 2022-06-07

**Authors:** Gunnar Husabø, Einar Hovlid

**Affiliations:** ^1^Department of Social Science, Western Norway University of Applied Sciences, Sogndal, Norway.; ^2^Department of Global Public Health and Primary Care, University of Bergen, Bergen, Norway.

**Keywords:** External Reviews, Equity, Justice, Supervision

## Abstract

Tama et al offer us an interesting analysis of a piloted regulatory reform that introduced a Joint Health Inspections (JHIs) system in three Kenyan counties. The study highlights key factors facilitating or hindering the implementation of the reform. In this commentary we reflect on the concept of fairness, which is one of the topics that is discussed in the study. We describe four important dimensions of fairness in the context of inspections: expectation clarity, consistency of assessment, consistency of enforcement, and fairness to patients. We argue that all four dimensions are important in the regulatory design, in order for the inspection to be perceived as fair.

 There has been a growing recognition of the importance of governance for healthcare quality in resource-limited settings, and for national policies that ensure sustainability of change and resilience of health systems.^1^ To that end, frameworks for inspections and other forms of external reviews are being employed in order to strengthen governance and improve quality and safety for patients.^2^ Though there are many different varieties of such frameworks, most include components of standard-setting, information-gathering, and behavior-modification, as described in a study by Hood and collegues.^3^

 Tama and colleagues’^4^ study of the pilot implementations of the Joint Health Inspection (JHI) in Kenyan health facilities offers a perspective that should be welcomed by the research community as a valuable addition to the literature on inspections. If we are to understand how, why, and when regulatory interventions work, we need to understand the contexts they operate within.^5^ The existing literature on regulation from resource limited countries has been sparse, and a vast majority of research on external reviews has been carried out in Western Europe, the United States, and Australia. We therefore clearly need more studies that can shed light on how inspections work in low- and middle-income countries (LMICs), and especially ones that account for barriers and constraints particular to the LMICs. Furthermore, our ideas of how inspections work likely rest on taken-for-granted assumptions regarding the health systems and societies that inspections take place in. By producing accounts that can be compared to those from higher-income settings, studies of inspections in LMICs can bring deeper insight into the phenomenon of inspections in itself and help us better understand which factors that are important in order to design more effective inspections.

 Seeking to identify factors that facilitate or hinder implementation, Tama et al interviewed staff from national, county, and facility level who had been involved in the JHI. Fairness in the inspection process emerges as one of the central themes of their study. Fairness was emphasized in the design of the JHI, and the staff reported that they generally felt the inspections to be fair, and more so than the previous inspection system.

 In and of itself, fairness is a laudable objective. Fairness is also important for the functioning of regulatory work. Unfairness is a common complaint whenever regulatory efforts are criticized, and the perception that inspections are unfair can preclude, among other things, voluntary compliance.^6^ But how should we think about fairness in the context of inspection work, and what does it take for inspections to be considered fair? Fairness is a complex and multifaceted concept. We take as our starting point that fairness requires acting impartially and that fair actions and judgements should adhere to the formal principle of equality: Differential treatment is only permissible when cases differ in ways relevant to the situation.^7^ Deciding what is relevant to the situation can of course be both difficult and contentious. As a practical contribution to moral reasoning and analysis in the context of inspections, we outline four dimensions of fairness. These dimensions are related to each of the three components of standard-setting, information-gathering, and behavior-modification.^3^ Additionally, recognizing that a main objective of inspections is the protection and improvement of public health and safety, we include fairness to the patients as a fourth dimension.

## Four Dimensions of Fairness

 One important dimension of fairness is expectation clarity. This is related to the component of standard-setting. The “rules of the game,” standards, responsibilities, and penalties should be clearly stated.^8^ The inspected organizations should know beforehand what criteria they might be judged against. This does not mean that the inspection criteria should be set in stone. The inspection authorities should be free to prioritize the theme of the inspection. If the inspection authorities always choose the same criteria or indicators, there is an increased risk of “gaming,” where the inspected organizations artificially boost the indicators prior to inspection either through directly falsification of numbers or through deliberate, often short-term, increased action that results in temporarily improved indicators that do not reflect an actual improvement in the overall care for the patients.^9^ However, the criteria chosen by the inspection authorities should be related to the stated goals for the sector, and these goals should be clearly communicated to the inspected organizations.

 Another dimension, this one related to information-gathering, is consistency of assessments. Similar cases should be treated similarly. In its most basic form, this requirement excludes instances of favoritism or bribery on the part of the inspection team. Challenges related to bribery are thoroughly discussed by Tama et al. Bribery is described as a “common feature” of the previous inspection system, where inspectors would solicit bribes in exchange for favorable outcomes of the inspections. Introducing mechanisms for transparency and accountability, the pilot inspection regime was perceived as by and large curbing bribery.

 More broadly, the inspection authorities should also be cognizant of how bias and chance variability can influence assessments. In some instances, the way the inspections are set up might systematically reward organizations that employ practices not actually related to the quality of their services. In other instances, inspectors let personal convictions influence their judgements, or random variability between inspectors’ assessments leads to similar cases being judged differently. Moving discretionary power from individuals to collectives and introducing quality assurance processes are examples of methods employed to ensure greater consistency in inspection judgements.^10^ The desire for reduced variability in assessments can, however, introduce dilemmas for the inspections authorities as they often encounter organizations in vastly different contexts when it comes to the population they serve and the resources they have available. Previous research into the ethical challenges of inspection work has noted how some inspectors are uneasy about issuing harsh judgements against organizations failing to meet standards when managers and professionals do their best under challenging conditions.^11^ The JHI encountered such dilemmas when they were met by criticism from public and some faith-based facilities that lacked the resources to comply with the guidelines. Even if the staff performed well under the circumstances, the facility might receive poor scores in the inspections.

 A third dimension is related to the way the inspection approaches behavior-modification: consistency of enforcement. Measures taken on the basis of assessments should be in accordance with the rules set out in relevant frameworks or regulations and similar infractions should evoke similar reactions. Moreover, once decided upon, measures should be carried out and followed up in accordance with the decision. This was noted as a potentially problematic issue in the case of the JHI, where some facilities that had been required to shut down were kept open because of political connections or the reluctance of county staff officers to enforce shutdowns in their own community.

 The fourth dimension of fairness in inspections that we want to highlight, is that of fairness to the patients. Frameworks for inspections are meant to serve the interests of patients and public health, the most important of which is healthcare quality. Being fair to patients means ensuring that patients receive high quality services and prioritizing issues that are directly or indirectly related to the quality of care. We should also recognize the importance of the patients’ perspectives on quality. Inspections often carry with them an implicit bias that favors the clinical perspectives of medical experts. Recent research has argued for developing regulatory approaches that are more open to the input of patients and their next of kin.^12^ This way of broadening the concept of quality introduces the dilemma of incommensurability between the inspectors’ and the patients’ views on what constitutes good quality can potentially complicate the assessment process.

 Moreover, sometimes there will be side effects of inspections that disadvantage the patients. Herein lies some potentially vexing dilemmas for the inspection authorities. In some instances, regulatory requirements need to take priority over the immediate interests of patients. A case in point is the potential negative impact of closure on access, which was one of the issues raised in Tama and colleagues’ study. A shutdown of a facility will mean the discontinuation of services for their patients. If they have no alternative to this facility, this could result in an unfair outcome for those patients even if all other aspects of the inspection have been conducted fairly.

## Balancing the Dimensions

 Though they partially overlap with items that can be found in “wish lists” of good regulation design,^2^ the dimensions we have outlined here are not meant as an exhaustive checklist for how to succeed with inspections. Fairness is only one facet of good regulation design, and in order to understand how to make inspections fair, it is important to also look to other, neighboring qualities, such as legitimacy, transparency, and competency.

 Of these, legitimacy is an overarching concern. The sources of legitimacy can be found throughout the whole inspection process, from input (including the legal basis for the inspections) to output (the substantive content of the inspection authorities’ assessments and reactions). Legitimacy can also depend on the throughput of the inspection,^13^ ie, the process by which the inspection authorities arrive at their conclusions and decisions. Transparency is especially important for bolstering throughput legitimacy. An inspection will not be perceived as fair without a certain degree of transparency into the process. In the JHIs, the facility staff received copies of the inspection checklist prior to inspection and summary reports at the end with suggestions for improvements. This was felt to increase confidence in the inspection process. We can expect transparency into the inspection process to influence attitudes not only in the health facilities, but also of the public in general, as providing information about why and how a health care regulator makes decisions has been shown to increase citizens’ trust in the regulator.^14^

 We would, however, caution against focusing on throughput legitimacy alone, ie, on issues such as expectation clarity and procedural fairness. This might lead to a form of proceduralism where legitimacy is reduced to the question of whether the procedures were followed, while the legal standing of the inspection authorities and the effects of the inspections are overlooked.^15^ Consistently carrying out inspections that are fair to the patients requires the inspection authorities to be informed and competent. In the JHI study, facility staff appreciated how the inspectors took time to explain what the facilities could do to improve. Clinical knowledge and knowledge of health systems are vital for the inspections to succeed, and the competency of inspectors is an important prerequisite for inspections to be considered legitimate.^16^

 Regulators should strive for a balanced fairness, designing inspections that engender trust in the process, ensure that facilities are safe, contribute to quality improvement, and are sensitive to the users’ perspectives on quality. This means that regulators need to engage with the dilemmas that inevitably will present themselves in the course of an inspection process.

## Ethical issues

 Not applicable.

## Competing interests

 Both authors declare that they have no competing interests.

## Authors’ contributions

 Conception and design: GH and EH; Draft: GH; Critical revision of the manuscript for important intellectual content: GH and EH.
